# Independent association of HLA-DPB1*02:*01* with rheumatoid arthritis in Japanese populations

**DOI:** 10.1371/journal.pone.0204459

**Published:** 2018-09-20

**Authors:** Hiroshi Furukawa, Shomi Oka, Kota Shimada, Atsushi Hashimoto, Akiko Komiya, Shinichiro Tsunoda, Akiko Suda, Satoshi Ito, Koichiro Saisho, Masao Katayama, Satoshi Shinohara, Takeo Sato, Katsuya Nagatani, Seiji Minota, Toshihiro Matsui, Naoshi Fukui, Shoji Sugii, Hajime Sano, Kiyoshi Migita, Shouhei Nagaoka, Shigeto Tohma

**Affiliations:** 1 Clinical Research Center for Allergy and Rheumatology, National Hospital Organization Sagamihara National Hospital, Sagamihara, Japan; 2 Molecular and Genetic Epidemiology Laboratory, Faculty of Medicine, University of Tsukuba, Tsukuba, Japan; 3 Department of Rheumatology, National Hospital Organization Sagamihara National Hospital, Sagamihara, Japan; 4 Department of Rheumatic Diseases, Tokyo Metropolitan Tama Medical Center, Fuchu, Japan; 5 Division of Rheumatology, Department of Internal Medicine, Hyogo College of Medicine, Nishinomiya, Japan; 6 Department of Rheumatology, Sumitomo Hospital, Osaka, Japan; 7 Department of Rheumatology, Yokohama Minami Kyosai Hospital, Yokohama, Japan; 8 Department of Rheumatology, Niigata Rheumatic Center, Shibata, Japan; 9 Department of Orthopedics/Rheumatology, Miyakonojo Medical Center, National Hospital Organization, Miyakonojo, Japan; 10 Department of Internal Medicine, Nagoya Medical Center, National Hospital Organization, Nagoya, Japan; 11 Tochigi Rheumatology Clinic, Utsunomiya, Japan; 12 Division of Rheumatology and Clinical Immunology, Jichi Medical University, Shimotsuke, Japan; 13 Clinical Research Center, Nagasaki Medical Center, National Hospital Organization, Omura, Japan; 14 Department of Gastroenterology and Rheumatology, Fukushima Medical University School of Medicine, 1 Hikarigaoka, Fukushima, Japan; 15 Tokyo National Hospital, National Hospital Organization, Kiyose, Japan; University of the Chinese Academy of Sciences, CHINA

## Abstract

**Objective:**

Rheumatoid arthritis (RA) is a chronic autoimmune disease characterized with joint destructions; environmental and genetic factors were thought to be involved in the etiology of RA. The production of anti-citrullinated peptide antibodies (ACPA) is specifically associated with RA. *DRB1* is associated with the susceptibility of RA, especially ACPA-positive RA [ACPA(+)RA]. However, a few studies reported on the independent associations of *DPB1* alleles with RA susceptibility. Thus, we investigated the independent association of *DPB1* alleles with RA in Japanese populations.

**Methods:**

Association analyses of *DPB1* were conducted by logistic regression analysis in 1667 RA patients and 413 controls.

**Results:**

In unconditioned analysis, DPB1*04:*02* was nominally associated with the susceptibility of ACPA(+)RA (*P* = 0.0021, corrected *P* (*Pc*) = 0.0275, odds ratio [OR] 1.52, 95% confidence interval [CI] 1.16–1.99). A significant association of DPB1*02:*01* with the susceptibility of ACPA(+)RA was observed, when conditioned on *DRB1* (*P*_adjusted_ = 0.0003, *P*c_adjusted_ = 0.0040, OR_adjusted_ 1.47, 95%CI 1.19–1.81). DPB1*05:*01* was tended to be associated with the protection against ACPA(+)RA, when conditioned on *DRB1* (*P*_adjusted_ = 0.0091, *P*c_adjusted_ = 0.1184, OR_adjusted_ 0.78, 95%CI 0.65–0.94). When conditioned on *DRB1*, the association of DPB1*04:*02* with ACPA(+)RA was disappeared. No association of *DPB1* alleles with ACPA-negative RA was detected.

**Conclusion:**

The independent association of DPB1*02:*01* with Japanese ACPA(+)RA was identified.

## Introduction

Rheumatoid arthritis (RA) is a systemic autoimmune disease characterized with synovial joint destructions and extra-articular manifestations. The etiology of RA is still unknown, but environmental and genetic factors were thought to be involved in the pathogenesis of RA [1,2,3]. Human leukocyte antigen (HLA) is the strongest genetic factor in RA and it was confirmed in genome wide association studies based on single nucleotide polymorphisms [4]. *DRB1* was believed to be the most important locus in *HLA* for the susceptibility of RA; some *DRB1* alleles were associated with the susceptibility of RA and have common motifs of amino acid residues at position 70–74 (QKRAA, RRRAA, or QRRAA) in DRβ chain [5]. These were designated as shared epitope (SE) alleles [6]. DRB1*04:*01* was mainly associated with RA in European populations [5] and DRB1*04:*05* in Asian [7]. Although both of DRB1*04:*01* and DRB1*04:*05* are SE alleles, these differences could be explained by the different frequencies of these susceptibility alleles for RA in different ethnic groups. The production of anti-citrullinated peptide antibodies (ACPA) is specifically associated with RA. ACPA-positive RA [ACPA(+)RA] is strongly associated with SE alleles, but ACPA-negative RA [ACPA(-)RA] is weakly [7,8,9].

Some reports also suggested that *B*, *DQB1*, or *DPB1* would be involved in the pathogenesis of RA [10,11,12,13,14,15,16,17,18,19,20]. Since *HLA* region is in strong linkage disequilibrium, it is important to eliminate the effects of *DRB1* to elucidate the role of other loci in *HLA*. The independent associations of amino acid residues in *B* and *DPB1* loci were recently reported [21,22,23]. However, few studies reported on the independent associations of *DPB1* alleles for RA susceptibility. Since *DRB1* is the strongest genetic risk factor for ACPA(+)RA, we investigated the independent association of *DPB1* alleles from *DRB1* in Japanese ACPA(+)RA.

## Materials and methods

### Patients

One thousand six hundred sixty seven Japanese RA patients were recruited at Hyogo College of Medicine, Jichi Medical University, Miyakonojo Medical Center, Nagasaki Medical Center, Nagoya Medical Center, Niigata Rheumatic Center, Sagamihara National Hospital, Tochigi Rheumatology Clinic, Tokyo Metropolitan Tama Medical Center, or Yokohama Minami Kyosai Hospital. RA patients fulfilled the 1987 American College of Rheumatology criteria for RA [24] or the 2010 Rheumatoid Arthritis Classification Criteria [25]. Four hundred thirteen Japanese healthy controls (mean age ± SD, 39.3 ± 11.0 years, vs. ACPA(+)RA: *P* = 6.50X10^-130^, vs. ACPA(-)RA, *P* = 5.51X10^-71^, 61 male [14.8%], vs. ACPA(+)RA: *P* = 0.0792, vs. ACPA(-)RA, *P* = 0.2195) were recruited at Kanazawa University, Sagamihara National Hospital, and Teikyo University [26] or by the Pharma SNP Consortium (Tokyo, Japan) [27,28]. Rheumatoid factor and ACPA were measured by N-latex RF kit (Siemens Healthcare Diagnostics, München, Germany) or Mesacup-2 test CCP (Medical & Biological Laboratories, Nagoya, Japan), respectively. This study was reviewed and approved by Hyogo College of Medicine Research Ethics Committee, Jichi Medical University Research Ethics Committee, Miyakonojo Medical Center Research Ethics Committee, Nagasaki Medical Center Research Ethics Committee, Nagoya Medical Center Research Ethics Committee, Niigata Rheumatic Center Research Ethics Committee, Sagamihara National Hospital Research Ethics Committee, Tokyo Metropolitan Tama Medical Center Research Ethics Committee, Yokohama Minami Kyosai Hospital Research Ethics Committee, and University of Tsukuba Research Ethics Committee. Written informed consent was obtained from all study participants. This study was conducted in accordance with the principles expressed in the Declaration of Helsinki.

### Genotyping of *DRB1* and *DPB1*

Genotyping of *DRB1* and *DPB1* was performed by polymerase chain reaction with reverse sequence-specific oligonucleotide probes (WAKFlow HLA typing kit, Wakunaga Pharmaceutical Co., Ltd., Akitakata, Japan) and Bio-Plex 200 (Bio-Rad, Hercules, CA). SE alleles contain DRB1*01:*01*, DRB1*04:*01*, DRB1*04:*05*, DRB1*04:*10*, DRB1*10:*01*, and DRB1*14:*06* [5]. Genotyping results for some of the RA patients and the healthy controls were previously reported [7,26,29,30,31,32].

### Statistical analysis

Clinical features of the RA patients were analyzed by Fisher’s exact test using 2X2 contingency tables or Student’s t-test. Unconditioned logistic regression analysis under the additive model was performed to analyze nominal associations of *HLA* alleles with the susceptibility of RA. On the other hand, conditioned logistic regression analysis was used to investigate the independent contribution of each *DPB1* allele from *DRB1* to the susceptibility of RA. *P*_adjusted_ and OR_adjusted_ were calculated for *DPB1* alleles, when conditioned on *DRB1*. Alleles detected in both case and control groups were tested. The two-locus analysis was also conducted by logistic regression analysis under the additive model to identify the primary role of associated *DRB1* or *DPB1* alleles. Haplotype frequencies of *DRB1-DPB1* were estimated with expectation-maximization algorithm with SNPAlyze ver.8.0.4 Pro (Dynacom, Chiba, Japan) and Permutation *P* values were established by 100000 permutations. Logistic regression analysis under the additive model was also performed to analyze associations of amino acid residues; conditional logistic regression analysis was used to investigate the independent contribution of each DPβ chain amino acid residue from DRβ chain amino acid residues to the susceptibility of RA. *P*_adjusted_ values were calculated for amino acid residues in the DPβ chains, when conditioned on DRβ chain amino acid residues. Multiple comparisons were adjusted by Bonferroni method; corrected *P* (*Pc*) values were derived from multiplying the *P* values by the number of alleles or amino acid residues tested.

## Results

### Clinical manifestations of RA patients

Characteristics of RA patients are shown in Table 1. Steinbrocker stage and class [33] were higher in ACPA(+)RA than ACPA(-)RA. The rheumatoid factor positivity rate was also higher.

**Table 1:** Characteristics of RA patients.

	ACPA(+)RA	ACPA(-)RA	*P*
Number	1436	231	
Mean age, years (SD)	62.8 (12.3)	61.8 (12.5)	*0.2991
Male, n (%)	267 (18.7)	43 (18.7)	1.0000
Age at onset (SD)	49.8 (14.3)	51.9 (16.0)	*0.0850
Steinbrocker stage III and IV, n (%)	629 (48.6)	61 (31.8)	1.26X10^-5^
Steinbrocker class 3 and 4, n (%)	195 (15.1)	15 (7.9)	0.0072
Rheumatoid factor positive, n (%)	1192 (88.8)	79 (37.4)	1.86X10^-56^

RA: rheumatoid arthritis, ACPA: anti-citrullinated peptide antibody, ACPA(+)RA: ACPA positive RA, ACPA(-)RA: ACPA negative RA. Association was tested between ACPA(+)RA and ACPA(-)RA by Fisher’s exact test using 2X2 contingency tables or Student’s t-test.

*Student’s t-test was employed.

### Association of *DPB1* with ACPA(+)RA

Association of *DRB1* with ACPA(+)RA was confirmed (S1 Table), as reported in the previous study [7]; DRB1*04:*05* and *04:*01* were associated with the susceptibility of ACPA(+)RA and DRB1*04:*06*, *08:*02*, *08:*03*, *13:*02*, and *14:*03* were protectively associated. Next, it was analyzed whether *DPB1* was also associated with ACPA(+)RA (Table 2). In unconditioned analysis, DPB1*04:*02* was nominally associated with the susceptibility of ACPA(+)RA (*P* = 0.0021, *P*c = 0.0275, odds ratio [OR] 1.52, 95% confidence interval [CI] 1.16–1.99, Table 2, left column). Since *DRB1* and *DPB1* are in strong linkage disequilibrium, nominal associations of *DPB1* alleles were influences by the associations of *DRB1* alleles with ACPA(+)RA. In order to clarify whether each *DPB1* allele was independently associated with ACPA(+)RA, conditional logistic regression analysis was performed (Table 2, right column). The significant association of DPB1*02:*01* with the susceptibility of ACPA(+)RA was observed, when conditioned on *DRB1* (*P*_adjusted_ = 0.0003, *P*c_adjusted_ = 0.0040, OR_adjusted_ 1.47, 95%CI 1.19–1.81, Table 2, right column). DPB1*05:*01* was tended to be associated with the protection against ACPA(+)RA, when conditioned on *DRB1* (*P*_adjusted_ = 0.0091, *P*c_adjusted_ = 0.1184, OR_adjusted_ 0.78, 95%CI 0.65–0.94, Table 2, right column). When conditioned on *DRB1*, DPB1*04:*02* was not associated with the susceptibility of ACPA(+)RA, suggesting the influence of *DRB1* on the nominal association of DPB1*04:*02*. Thus, DPB1*02:*01* was independently associated with the susceptibility of ACPA(+)RA.

**Table 2:** Conditional logistic regression analysis of *DPB1* alleles in ACPA(+) RA and controls.

			Uncoditioned				Conditioned on *DRB1*			
DPB1 allele	ACPA(+)RA (2n = 2872)	Control (2n = 826)	OR	95%CI	*P*	*P*c	OR_adjusted_	95%CI	*P*_adjusted_	*Pc*_adjusted_
DPB1*02:*01*	781 (27.2)	198 (24.0)	1.19	(0.99–1.42)	0.0644	0.8371	1.47	(1.19–1.81)	0.0003	0.0040
DPB1*02:*02*	119 (4.1)	34 (4.1)	1.01	(0.68–1.49)	0.9723	NS	1.09	(0.69–1.72)	0.6998	NS
DPB1*03:*01*	113 (3.9)	36 (4.4)	0.91	(0.62–1.33)	0.6150	NS	0.80	(0.52–1.24)	0.3141	NS
DPB1*04:*01*	98 (3.4)	44 (5.3)	0.64	(0.45–0.92)	0.0147	0.1909	1.00	(0.58–1.72)	0.9912	NS
DPB1*04:*02*	351 (12.2)	69 (8.4)	1.52	(1.16–1.99)	0.0021	0.0275	1.12	(0.79–1.58)	0.5362	NS
DPB1*05:*01*	1054 (36.7)	319 (38.6)	0.92	(0.78–1.08)	0.3114	NS	0.78	(0.65–0.94)	0.0091	0.1184
DPB1*06:*01*	15 (0.5)	3 (0.4)	1.44	(0.42–5.01)	0.5638	NS	2.15	(0.56–8.22)	0.2640	NS
DPB1*09:*01*	218 (7.6)	87 (10.5)	0.70	(0.54–0.91)	0.0072	0.0930	0.67	(0.40–1.10)	0.1132	NS
DPB1*13:*01*	36 (1.3)	19 (2.3)	0.53	(0.30–0.94)	0.0297	0.3862	0.57	(0.31–1.06)	0.0782	NS
DPB1*14:*01*	44 (1.5)	8 (1.0)	1.60	(0.75–3.43)	0.2262	NS	1.06	(0.45–2.48)	0.9019	NS
DPB1*17:*01*	6 (0.2)	3 (0.4)	0.57	(0.14–2.30)	0.4330	NS	0.49	(0.05–5.05)	0.5478	NS
DPB1*19:*01*	13 (0.5)	2 (0.2)	1.88	(0.42–8.35)	0.4082	NS	1.08	(0.23–5.08)	0.9216	NS
DPB1*41:*01*	7 (0.2)	2 (0.2)	1.01	(0.21–4.86)	0.9934	NS	0.87	(0.14–5.48)	0.8826	NS

RA: rheumatoid arthritis, ACPA: anti-citrullinated peptide antibody, ACPA(+)RA: ACPA positive RA, OR: odds ratio, CI: confidence interval, *P*c: corrected *P* value, NS: not significant. Allele frequencies are shown in parenthesis (%).The association of each *DPB1* allele with ACPA(+)RA was analyzed by logistic regression analysis. The left column indicates the results from unconditioned analyses. The right column indicates the results from analyses conditioned on *DRB1*. *P*_adjusted_ and OR_adjusted_ were calculated by conditional logistic regression analysis under the additive model. Corrected *P* (*P*c) values were calculated by multiplying the *P* value by the number of alleles tested.

In order to reveal whether each *DRB1* allele influenced on the association of DPB1*02:*01* with the susceptibility of ACPA(+)RA, conditional logistic regression analysis was conducted (Table 3). When conditioned on DRB1*04:*05*, the significant association of DPB1*02:*01* with the susceptibility of ACPA(+)RA was observed (*P*_adjusted_ = 0.0073, OR_adjusted_ 1.29, 95%CI 1.07–1.56, Table 3). Because DRB1*04:*05* is the strongest risk factor for RA in Asian [7], the influence of DRB1*04:*05* on the nominal association of DPB1*02:*01* would be strongest. However, the stronger association of DPB1*02:*01* with the susceptibility of ACPA(+)RA was observed, when conditioned on SE alleles (*P*_adjusted_ = 0.0016, OR_adjusted_ 1.37, 95%CI 1.13–1.66, Table 3) or *DRB1* (*P*_adjusted_ = 0.0003, OR_adjusted_ 1.47, 95%CI 1.19–1.81, Table 3). These data suggested that many *DRB1* alleles including DRB1*04:*05* had influenced on the nominal association of DPB1*02:*01* with the susceptibility of ACPA(+)RA.

**Table 3:** Conditional logistic regression analysis of DPB1*02:*01* between ACPA(+) RA and controls.

Uncoditioned			
	OR	95%CI	*P*
	1.19	(0.99–1.42)	0.0644
Conditioned on each *DRB1* allele			
	OR_adjusted_	95%CI	*P*_adjusted_
DRB1*01:*01*	1.22	(1.02–1.47)	0.0307
DRB1*04:*01*	1.17	(0.97–1.40)	0.0934
DRB1*04:*03*	1.20	(1.00–1.43)	0.0507
DRB1*04:*05*	1.29	(1.07–1.56)	0.0073
DRB1*04:*06*	1.25	(1.04–1.51)	0.0165
DRB1*04:*07*	1.19	(0.99–1.42)	0.0590
DRB1*04:*10*	1.19	(0.99–1.42)	0.0596
DRB1*07:*01*	1.18	(0.99–1.42)	0.0655
DRB1*08:*02*	1.22	(1.01–1.46)	0.0359
DRB1*08:*03*	1.20	(1.00–1.43)	0.0520
DRB1*09:*01*	1.18	(0.98–1.41)	0.0740
DRB1*10:*01*	1.17	(0.98–1.40)	0.0844
DRB1*11:*01*	1.19	(0.99–1.42)	0.0596
DRB1*12:*01*	1.18	(0.98–1.41)	0.0776
DRB1*12:*02*	1.19	(0.99–1.42)	0.0644
DRB1*13:*01*	1.20	(1.00–1.44)	0.0454
DRB1*13:*02*	1.16	(0.97–1.39)	0.1052
DRB1*14:*03*	1.21	(1.01–1.46)	0.0366
DRB1*14:*05*	1.18	(0.98–1.41)	0.0727
DRB1*14:*06*	1.19	(0.99–1.42)	0.0625
DRB1*14:*07*	1.18	(0.99–1.42)	0.0664
DRB1*14:*54*	1.18	(0.98–1.41)	0.0733
DRB1*15:*01*	1.21	(1.01–1.46)	0.0373
DRB1*15:*02*	1.16	(0.96–1.39)	0.1171
DRB1*16:*02*	1.19	(0.99–1.42)	0.0628
SE	1.37	(1.13–1.66)	0.0016
*DRB1*	1.47	(1.19–1.81)	0.0003

RA: rheumatoid arthritis, ACPA: anti-citrullinated peptide antibody, ACPA(+)RA: ACPA positive RA, OR: odds ratio, CI: confidence interval, SE: Shared epitope. The association of DPB1*02:*01* with ACPA(+) RA was analyzed by logistic regression analysis. The first row indicates the results from unconditioned analyses. The other rows indicate the results from analyses conditioned on shown *DRB1* alleles. *P*_adjusted_ and OR_adjusted_ were calculated by conditional logistic regression analysis under the additive model.

The two-locus analysis was conducted to identify the primary role of DRB1*04:*05* and DPB1*02:*01* for the susceptibility of ACPA(+)RA (S2 Table). The OR for DPB1*02:*01* in ACPA(+)RA patients with DRB1*04:*05* was 1.45 (*P* = 0.0688, S2 Table), while the OR for DPB1*02:*01* in ACPA(+)RA patients without DRB1*04:*05* was 1.26 (*P* = 0.0356, S2 Table). On the other hand, the OR for DRB1*04:*05* in ACPA(+)RA patients with DPB1*02:*01* was 4.21 (*P* = 3.71X10^-12^, S2 Table), and the OR for DRB1*04:*05* in ACPA(+)RA patients without DPB1*02:*01* was 3.33 (*P* = 7.58X10^-15^, S2 Table). These results suggested the independent roles of DRB1*04:*05* and DPB1*02:*01* on the susceptibility of ACPA(+)RA.

When haplotype frequencies were compared between ACPA(+)RA patients and controls, three haplotypes including DRB1*04:*05* were associated with the susceptibility of ACPA(+)RA (DRB1*04:05-DPB1*02:*01*; Permutation *P*<0.0001, DRB1*04:05-DPB1*04:*02*,; Permutation *P* = 0.0004, DRB1*04:05-DPB1*05:*01*; Permutation *P*<0.0001, S3 Table), suggesting the primary role of DRB1*04:*05*. On the other hand, some haplotypes including DPB1*02:*01* were associated with the ACPA(+)RA susceptibility (DRB1*04:05-DPB1*02:*01*; Permutation *P* <0.0001, DRB1*09:01-DPB1*02:*01*; Permutation *P* = 0.0048, S3 Table) or the protection (DRB1*04:06-DPB1*02:*01*; Permutation *P* = 0.0149, DRB1*08:02-DPB1*02:*01*; Permutation *P*<0.0001, DRB1*13:02-DPB1*02:*01*; Permutation *P* = 0.0015, DRB1*15:01-DPB1*02:*01*; Permutation *P* = 0.0208, S3 Table), suggesting the influences of *DRB1* alleles on the effects of DPB1*02:*01*. These data suggested the stronger effects of *DRB1* alleles on the ACPA(+)RA susceptibility or the protection.

### Association of *DPB1* with ACPA(-)RA

It was analyzed whether *DPB1* was also associated with ACPA(-)RA (Table 4). In unconditioned analysis, no *DPB1* allele was associated with the susceptibility of ACPA(-)RA (Table 4, left column). In order to elucidate whether each *DPB1* allele was independently associated with ACPA(-)RA, conditional logistic regression analysis was performed (Table 4, right column). No association of *DPB1* alleles with the susceptibility of ACPA(-)RA was observed, when conditioned on *DRB1*. Association of *DPB1* was also analyzed with overall RA (S4 Table). In unconditioned analysis, no *DPB1* allele was associated with the overall RA (S4 Table, left column). When conditioned on *DRB1*, DPB1*02:*01* was associated with the overall RA (S4 Table, right column). However, the association was weaker than ACPA(+)RA.

**Table 4:** Conditional logistic regression analysis of *DPB1* alleles between ACPA(-)RA and controls.

			Uncoditioned				Conditioned on *DRB1*			
DPB1 allele	ACPA(-)RA (2n = 462)	Control (2n = 826)	OR	95%CI	*P*	*P*c	OR_adjusted_	95%CI	*P*_adjusted_	*Pc*_adjusted_
DPB1*02:*01*	118 (25.5)	198 (24.0)	1.09	(0.84–1.42)	0.5270	NS	1.20	(0.90–1.60)	0.2238	NS
DPB1*02:*02*	12 (2.6)	34 (4.1)	0.62	(0.32–1.21)	0.1645	NS	0.64	(0.31–1.33)	0.2277	NS
DPB1*03:*01*	21 (4.5)	36 (4.4)	1.05	(0.60–1.82)	0.8750	NS	0.86	(0.47–1.58)	0.6332	NS
DPB1*04:*01*	19 (4.1)	44 (5.3)	0.77	(0.45–1.32)	0.3456	NS	0.77	(0.36–1.64)	0.4931	NS
DPB1*04:*02*	42 (9.1)	69 (8.4)	1.10	(0.73–1.65)	0.6486	NS	0.88	(0.53–1.46)	0.6201	NS
DPB1*05:*01*	190 (41.1)	319 (38.6)	1.10	(0.88–1.39)	0.3891	NS	1.00	(0.77–1.30)	0.9834	NS
DPB1*06:*01*	3 (0.6)	3 (0.4)	1.80	(0.36–8.98)	0.4746	NS	1.69	(0.31–9.30)	0.5491	NS
DPB1*09:*01*	36 (7.8)	87 (10.5)	0.72	(0.48–1.08)	0.1137	NS	0.88	(0.44–1.74)	0.7097	NS
DPB1*13:*01*	10 (2.2)	19 (2.3)	0.94	(0.43–2.05)	0.8734	NS	0.94	(0.41–2.15)	0.8856	NS
DPB1*14:*01*	6 (1.3)	8 (1.0)	1.35	(0.46–3.94)	0.5829	NS	1.47	(0.44–4.98)	0.5326	NS
DPB1*17:*01*	1 (0.2)	3 (0.4)	0.59	(0.06–5.75)	0.6530	NS	2.07	(0.09–47.37)	0.6493	NS
DPB1*41:*01*	3 (0.6)	2 (0.2)	2.70	(0.45–16.30)	0.2778	NS	2.43	(0.37–15.87)	0.3554	NS

RA: rheumatoid arthritis, ACPA: anti-citrullinated peptide antibody, ACPA(-)RA: ACPA negative RA, OR: odds ratio, CI: confidence interval, *P*c: corrected *P* value, NS: not significant. Allele frequencies are shown in parenthesis (%). Association was tested between ACPA(-) RA and controls by logistic regression analysis. *P*_adjusted_ and OR_adjusted_ were calculated by conditional logistic regression analysis under the additive model. Corrected *P* (*P*c) values were calculated by multiplying the *P* value by the number of alleles tested.

### Association of DPβ chain amino acid residues with ACPA(+)RA

**Fig 1:**
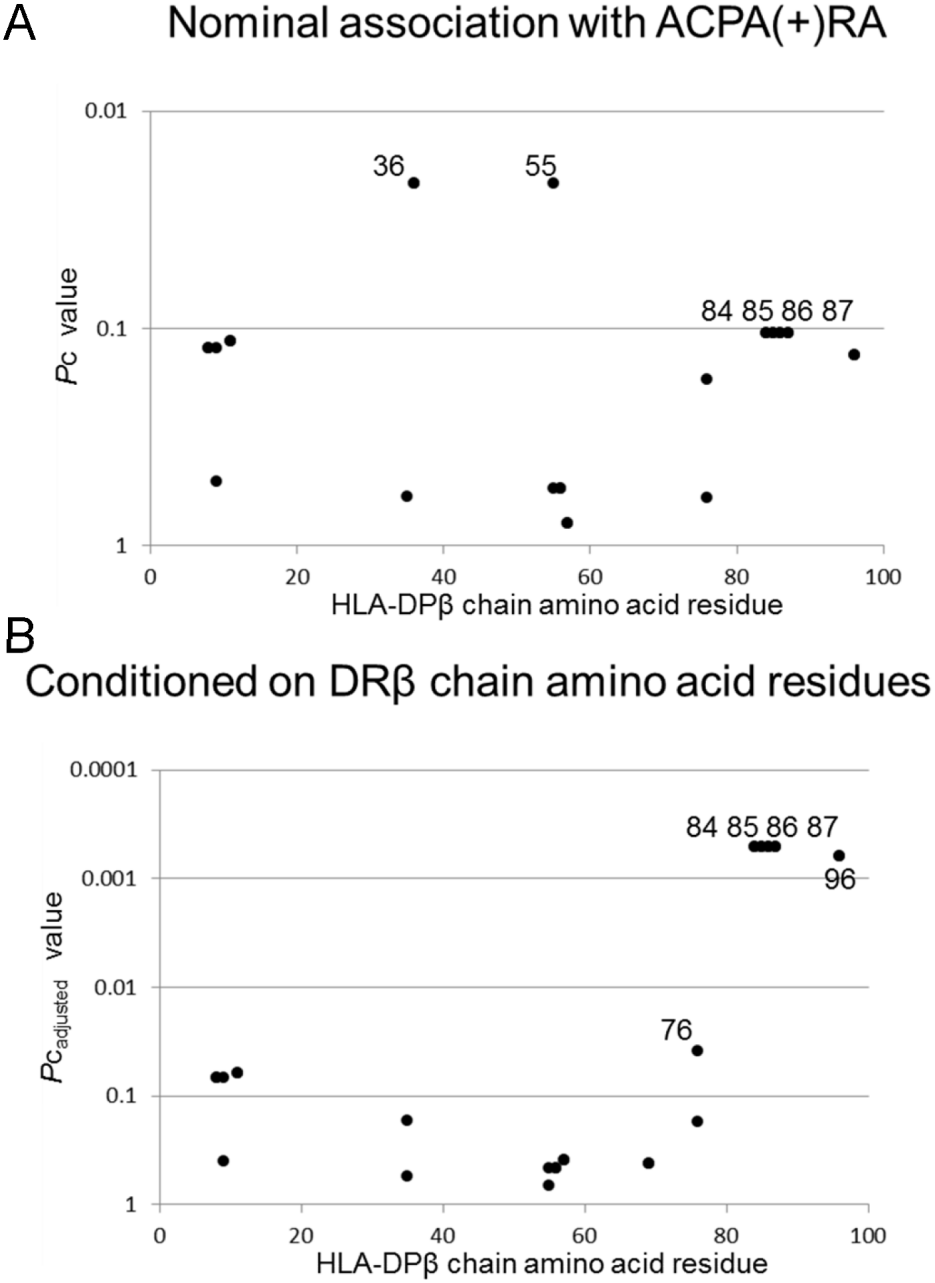
Associations of amino acid residues in the DPβ chains with ACPA(+)RA. **(A)** Association was established between ACPA(+)RA and controls by logistic regression analysis. **(B)** Conditional logistic regression analysis was performed to clarify whether each DPβ chain amino acid residue was independently associated with ACPA(+)RA. *P*_adjusted_ values were calculated for amino acid residues in the DPβ chains, when conditioned on DRβ chain amino acid residues. Corrected *P* (*P*c) values were obtained by multiplying the *P* value by the number of amino acid residues tested. RA: rheumatoid arthritis, ACPA: anti-citrullinated peptide antibody, ACPA(+)RA: ACPA positive RA.

Association of DRβ chain amino acid residues with ACPA(+)RA was confirmed (S1 Fig), as reported in the previous study [7]; 10Y, 11S, 12T, 13H, 33N, 70D, 96Y, and 98K in the DRβ chain showed associations. Two amino acid residues, 36A 55A, in the DPβ chain were slightly associated with ACPA(+)RA in unconditioned analysis (Fig 1A). In order to clarify whether each DPβ chain amino acid residue was independently associated with ACPA(+)RA, conditional logistic regression analysis was performed. Five amino acid residues, 84G (*P* = 3.20X10^-5^, *P*c = 0.0005, OR = 1.48, 95% CI 1.23–1.79), 85G (*P* = 3.20X10^-5^, *P*c = 0.0005, OR = 1.48, 95% CI 1.23–1.79), 86P (*P* = 3.20X10^-5^, *P*c = 0.0005, OR = 1.48, 95% CI 1.23–1.79), 87M (*P* = 3.20X10^-5^, *P*c = 0.0005, OR = 1.48, 95% CI 1.23–1.79), and 96R (*P* = 3.94X10^-5^, *P*c = 0.0006, OR = 1.48, 95% CI 1.23–1.78), in the DPβ chain were significantly associated with ACPA(+)RA, when conditioned on DRβ chain amino acid residues (Fig 1B). Since there are three haplotypes of these amino acid residues in the DPβ chain (84G-85G-86P-87M-96R, 84D-85E-86A-87V-96K, 84D-85E-86A-87V-96R), the results might reflect the effects of the haplotype of 84G-85G-86P-87M-96R on the susceptibility of ACPA(+)RA. The haplotype was actually associated with ACPA(+)RA in unconditioned analysis (*P* = 0.0078, OR = 1.24, 95% CI 1.06–1.44), or when conditioned on DRβ chain amino acid residues (*P*_adjusted_ = 4.11X10^-5^, OR_adjusted_ 1.47, 95%CI 1.22–1.77).

## Discussion

Although many investigations on the associations of *DRB1* alleles with the susceptibility of RA were performed, relatively fewer studies on the genetic effects of *DPB1* alleles for RA were conducted. There are a few direct reports on the independent association of *DPB1* alleles for the susceptibility of RA, though results of some studies suggested the role of *DPB1* alleles for RA [10,13,14,16,18]. DPB1*03:*01* was associated with rheumatoid factor negative RA in European descent [10]. When arginine at position 71 of DRβ chain was possessed, DPB1*04:*01* was associated with European RA [13]. DPB1*02:*01* and DPB1*06:*01* were associated with European RA without SE, whereas DPB1*04:*01* was associated with European RA with SE [14]. DPB1*02:*01* was associated with Japanese RA without DRB1*04:*05* [16]. The association of DPB1*02:*01* with the susceptibility of Japanese ACPA(+)RA and that of DPB1*04:*01*and DPB1*09:*01* with the protection were suggested [18]. However, the results of these previous studies could not conclude the independent association of DPB1*02:*01* from *DRB1*. In the present study, we directly revealed it in Japanese populations.

It was reported that phenylalanine at position 9 in DPβ chain was reported to be independently associated with European ACPA(+)RA [21]. Glycine at position 84 in DPβ chain was also independently associated with Japanese ACPA(+)RA [23]. However, the independently associated *DPB1* alleles were not reported in these studies. In the present study, glycine at position 84 in DPβ chain was independently associated with ACPA(+)RA and 84G-85G-86P-87M-96R in DPβ chain was the ACPA(+)RA susceptible haplotype. This ACPA(+)RA associated haplotype was included in DPB1*02:*01*, DPB1*02:*02*, DPB1*04:*01*, DPB1*04:*02*, and DPB1*41:*01*. Since the alleles other than DPB1*02:*01* were not directly associated with ACPA(+)RA susceptibility (Table 2, right column), DPB1*02:*01* would be mainly contributed to the risk of ACPA(+)RA among them. The independent association of phenylalanine at position 9 in DPβ chain was not confirmed in the present study, though this amino acid residue was included in DPB1*02:*01* and other alleles. This could be explained by the different distribution of *HLA* alleles in other ethnic populations. The amino acid residues 84, 85, 86, and 87 form the pocket 1 of DP peptide-binding groove [34]. This information suggested the involvement of peptides loaded on DP2 in the generation of ACPA or rheumatoid factor.

The association of DPB1*02:*01* with ACPA(-)RA was not detected in the present study (Table 4), because of the limited sample size of ACPA(-)RA. Although the association of *DPB1*with ACPA(-)RA was not found in the study on European populations [22], weak association was shown around *DP* loci in the other study on Japanese populations [23]. Therefore, this could be detected in future large scale studies. The independent association of DPB1*02:*01* with ACPA(+)RA should be replicated in future studies in Japanese populations and should be also analyzed in other populations. It was the limitation of this study that the population stratification was not excluded [35,36]. Thus, the present study revealed the independent association of DPB1*02:*01* with ACPA(+)RA in Japanese populations.

## Supporting information

S1 Fig Associations of amino acid residues in the DRβ chains with ACPA(+)RA. Association was established between ACPA(+)RA and controls by logistic regression analysis. Corrected P (Pc) values were obtained by multiplying the P value by the number of amino acid residues tested. RA: rheumatoid arthritis, ACPA: anti-citrullinated peptide antibody, ACPA(+)RA: ACPA positive RA.
(PDF)

S1 Table Logistic regression analysis of *DRB1* alleles in ACPA(+) RA and controls. RA: rheumatoid arthritis, ACPA: anticitrullinated peptide antibody, ACPA(+)RA: ACPA positive RA, OR: odds ratio, CI: confidence interval, P c: corrected P value, NS: not significant. Allele frequencies are shown in parenthesis (%). Association was tested by logistic regression analysis.
(PDF)

S2 Table Logistic regression analysis in the ACPA(+)RA patients and controls with or without DRB1*04:*05* or DPB1*02:*01*. RA: rheumatoid arthritis, ACPA: anti-citrullinated peptide antibody, ACPA(+)RA: ACPA positive RA, OR: odds ratio, CI: confidence interval. Association was tested between the RA patients and the controls with or without DRB1*04:*05* or DPB1*02:*01* by logistic regression analysis.
(PDF)

S3 Table *DRB1-DPB1* haplotype frequency in the ACPA(+)RA patients and controls. RA: rheumatoid arthritis, ACPA: anti-citrullinated peptide antibody, ACPA(+)RA: ACPA positive RA. Haplotypes with more than 1% frequency in controls are shown.
(PDF)

S4 Table Conditional logistic regression analysis of *HLA-DPB1* alleles in the RA patients and controls. RA: rheumatoid arthritis, OR: odds ratio, CI: confidence interval. Association was tested between the RA patients and the controls by Logistic regression analysis.
(PDF)
