# Supplementary material for: Independent association of HLA-DPB1*02:01 with rheumatoid arthritis in Japanese populations
Source: PLoS One. 2018 Sep 20;13(9):e0204459. doi: 10.1371/journal.pone.0204459 (PMC6157818; doi:10.1371/journal.pone.0204459)
Supplement: S1 Fig — Association was established between ACPA(+)RA and controls by logistic regression analysis. Corrected P (Pc) values were obtained by multiplying the P value by the number of amino acid residues tested. RA: rheumatoid arthritis, ACPA: anti-citrullinated peptide antibody, ACPA(+)RA: ACPA positive RA. (PDF) [file pone.0204459.s001.pdf]

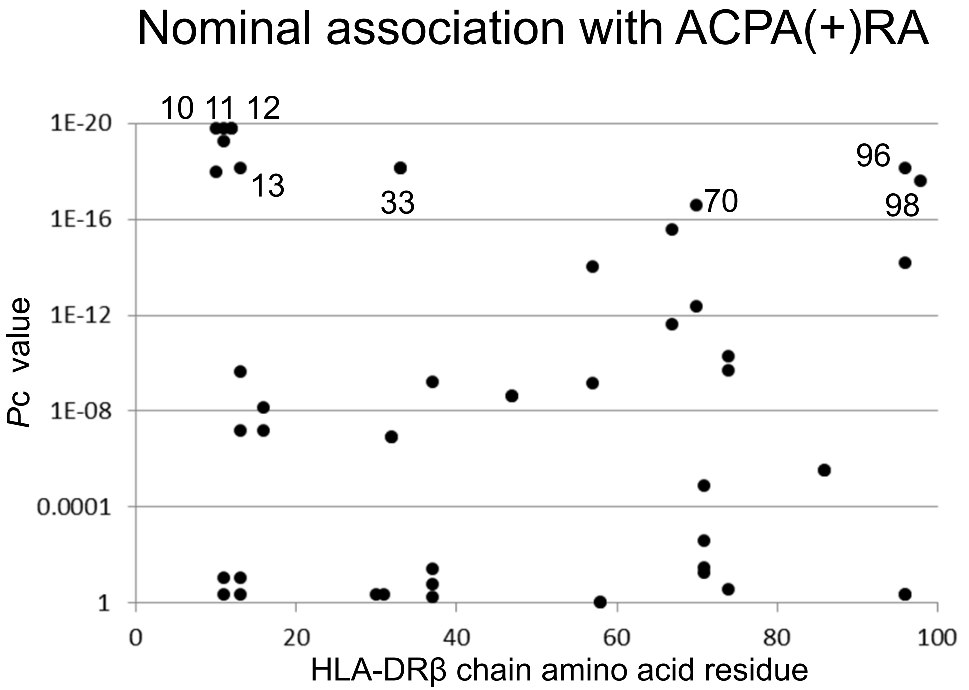

Supplementary Figure 1. Associations of amino acid residues in the DR β chains with ACPA(+)RA. Association was established between ACPA(+)RA and controls by logistic regression analysis. Corrected *P* (*P*c) values were obtained by multiplying the *P* value by the number of amino acid residues tested. RA: rheumatoid arthritis, ACPA: anti-citrullinated peptide antibody, ACPA(+)RA: ACPA positive RA..
