# Supplementary material for: Independent association of HLA-DPB1*02:01 with rheumatoid arthritis in Japanese populations
Source: PLoS One. 2018 Sep 20;13(9):e0204459. doi: 10.1371/journal.pone.0204459 (PMC6157818; doi:10.1371/journal.pone.0204459)
Supplement: S1 Table — RA: rheumatoid arthritis, ACPA: anticitrullinated peptide antibody, ACPA(+)RA: ACPA positive RA, OR: odds ratio, CI: confidence interval, P c: corrected P value, NS: not significant. Allele frequencies are shown in parenthesis (%). Association was tested by logistic regression analysis. (PDF) [file pone.0204459.s002.pdf]

S1 Table. Logistic regression analysis of *DRB1* alleles in ACPA(+) RA and controls.

	ACPA(+)RA (2n=2872)	Control (2n=826)	*OR*	95%CI	*P*	*P*c
*DRB1*01:01*	225 (7.8)	44 (5.3)	1.51	(1.08-2.11)	0.0152	0.3809
*DRB1*04:01*	102 (3.6)	8 (1.0)	3.70	(1.80-7.62)	0.0004	0.0097
*DRB1*04:03*	40 (1.4)	19 (2.3)	0.62	(0.36-1.05)	0.0775	NS
*DRB1*04:05*	836 (29.1)	94 (11.4)	3.55	(2.79-4.52)	$5.04X10^{-25}$	$1.26X10^{-23}$
*DRB1*04:06*	53 (1.8)	34 (4.1)	0.44	(0.28-0.68)	0.0002	0.0056
*DRB1*04:07*	5 (0.2)	3 (0.4)	0.48	(0.11-2.01)	0.3129	NS
*DRB1*04:10*	65 (2.3)	14 (1.7)	1.35	(0.75-2.43)	0.3158	NS
*DRB1*07:01*	8 (0.3)	3 (0.4)	0.77	(0.20-2.90)	0.6942	NS
*DRB1*08:02*	43 (1.5)	38 (4.6)	0.30	(0.19-0.48)	$2.40X10^{-7}$	$6.00X10^{-6}$
*DRB1*08:03*	118 (4.1)	64 (7.7)	0.53	(0.39-0.72)	$5.30X10^{-5}$	0.0013
*DRB1*09:01*	433 (15.1)	117 (14.2)	1.07	(0.86-1.33)	0.5285	NS
*DRB1*10:01*	28 (1.0)	2 (0.2)	3.87	(0.93-16.12)	0.0628	NS
*DRB1*11:01*	44 (1.5)	22 (2.7)	0.56	(0.33-0.95)	0.0310	0.7748
*DRB1*12:01*	81 (2.8)	32 (3.9)	0.73	(0.49-1.10)	0.1340	NS
*DRB1*12:02*	38 (1.3)	11 (1.3)	0.99	(0.52-1.91)	0.9853	NS
*DRB1*13:01*	2 (0.1)	5 (0.6)	0.11	(0.02-0.59)	0.0096	0.2388
*DRB1*13:02*	103 (3.6)	62 (7.5)	0.48	(0.35-0.66)	$5.44X10^{-6}$	0.0001
*DRB1*14:03*	29 (1.0)	21 (2.5)	0.40	(0.23-0.70)	0.0014	0.0359
*DRB1*14:05*	26 (0.9)	14 (1.7)	0.53	(0.27-1.02)	0.0558	NS
*DRB1*14:06*	46 (1.6)	16 (1.9)	0.83	(0.47-1.46)	0.5122	NS
*DRB1*14:07*	2 (0.1)	1 (0.1)	0.57	(0.05-6.35)	0.6513	NS
*DRB1*14:54*	68 (2.4)	28 (3.4)	0.70	(0.45-1.08)	0.1084	NS
*DRB1*15:01*	188 (6.5)	73 (8.8)	0.73	(0.56-0.97)	0.0276	0.6891
*DRB1*15:02*	254 (8.8)	92 (11.1)	0.77	(0.60-0.99)	0.0451	NS
*DRB1*16:02*	22 (0.8)	5 (0.6)	1.27	(0.48-3.37)	0.6321	NS

RA: rheumatoid arthritis, ACPA: anti-citrullinated peptide antibody, ACPA(+)RA: ACPA positive RA, OR: odds ratio, CI: confidence interval, *P*c: corrected *P* value, NS: not significant. Allele frequencies are shown in parenthesis (%). Association was tested by logistic regression analysis.
