# Supplementary material for: Independent association of HLA-DPB1*02:01 with rheumatoid arthritis in Japanese populations
Source: PLoS One. 2018 Sep 20;13(9):e0204459. doi: 10.1371/journal.pone.0204459 (PMC6157818; doi:10.1371/journal.pone.0204459)
Supplement: S2 Table — RA: rheumatoid arthritis, ACPA: anti-citrullinated peptide antibody, ACPA(+)RA: ACPA positive RA, OR: odds ratio, CI: confidence interval. Association was tested between the RA patients and the controls with or without DRB1*04:05 or DPB1*02:01 by logistic regression analysis. (PDF) [file pone.0204459.s003.pdf]

S2 Table. Logistic regression analysis in the ACPA(+)RA patients and controls with or without *DRB1*04:05* or *DPB1*02:01.*

*DPB1*02:01*	*P*	OR	(95%CI)
RA patients with *DRB1*04:05* vs. Controls with *DRB1*04:05*	0.0688	1.45	(0.97–2.16)
RA patients without *DRB1*04:05* vs. Controls without *DRB1*04:05*	0.0356	1.26	(1.02–1.56)
*DRB1*04:05*			
RA patients with *DPB1*02:01* vs. Controls with *DPB1*02:01*	$3.71X10^{-12}$	4.21	(2.81–6.31)
RA patients without *DPB1*02:0*1 vs. Controls without *DPB1*02:01*	$7.58X10^{-15}$	3.33	(2.46–4.51)

RA: rheumatoid arthritis, ACPA: anti–citrullinated peptide antibody, ACPA(+)RA: ACPA positive RA, OR: odds ratio, CI: confidence interval. Association was tested between the RA patients and the controls with or without *DRB1*04:05* or *DPB1*02:01* by logistic regression analysis.
