# Supplementary material for: Independent association of HLA-DPB1*02:01 with rheumatoid arthritis in Japanese populations
Source: PLoS One. 2018 Sep 20;13(9):e0204459. doi: 10.1371/journal.pone.0204459 (PMC6157818; doi:10.1371/journal.pone.0204459)
Supplement: S3 Table — RA: rheumatoid arthritis, ACPA: anti-citrullinated peptide antibody, ACPA(+)RA: ACPA positive RA. Haplotypes with more than 1% frequency in controls are shown. (PDF) [file pone.0204459.s004.pdf]

S3 Table. *DRB1-DPB1* haplotype frequency in the ACPA(+)RA patients and controls.

*DRB1**	*DPB1**	ACPA(+)RA	Control	Permutation *P*
*DRB1*04:05*	*DPB1*05:01*	0.15040	0.07140	<0.0001
*DRB1*08:02*	*DPB1*02:01*	0.00479	0.02070	<0.0001
*DRB1*04:05*	*DPB1*02:01*	0.06730	0.01400	<0.0001
*DRB1*08:02*	*DPB1*05:01*	0.00464	0.02070	0.0001
*DRB1*04:05*	*DPB1*04:02*	0.03780	0.01470	0.0004
*DRB1*13:02*	*DPB1*04:01*	0.02250	0.04600	0.0007
*DRB1*13:02*	*DPB1*02:01*	0.00395	0.01470	0.0015
*DRB1*11:01*	*DPB1*05:01*	0.00402	0.01440	0.0026
*DRB1*08:03*	*DPB1*02:02*	0.00894	0.02300	0.0030
*DRB1*09:01*	*DPB1*02:01*	0.06630	0.03980	0.0048
*DRB1*04:03*	*DPB1*05:01*	0.00286	0.01120	0.0054
*DRB1*04:06*	*DPB1*05:01*	0.00360	0.01220	0.0060
*DRB1*15:02*	*DPB1*09:01*	0.06770	0.09270	0.0080
*DRB1*04:06*	*DPB1*02:01*	0.01250	0.02560	0.0149
*DRB1*13:02*	*DPB1*05:01*	0.00403	0.01200	0.0170
*DRB1*15:01*	*DPB1*02:01*	0.02660	0.04290	0.0208
*DRB1*12:01*	*DPB1*05:01*	0.01250	0.02280	0.0340
*DRB1*01:01*	*DPB1*04:02*	0.05720	0.03950	0.0535
*DRB1*14:54*	*DPB1*05:01*	0.00735	0.01450	0.0597
*DRB1*08:03*	*DPB1*05:01*	0.01710	0.02840	0.0610
*DRB1*04:03*	*DPB1*02:01*	0.00503	0.01060	0.0752
*DRB1*14:05*	*DPB1*05:01*	0.00799	0.01450	0.1020
*DRB1*15:01*	*DPB1*05:01*	0.02800	0.03840	0.1096
*DRB1*08:03*	*DPB1*02:01*	0.01390	0.02040	0.1987
*DRB1*09:01*	*DPB1*05:01*	0.06930	0.07890	0.3579
*DRB1*15:02*	*DPB1*05:01*	0.00862	0.01200	0.4148

RA: rheumatoid arthritis, ACPA: anti-citrullinated peptide antibody, ACPA(+)RA: ACPA positive RA. Haplotypes with more than 1% frequency in controls are shown.
