# Supplementary material for: Independent association of HLA-DPB1*02:01 with rheumatoid arthritis in Japanese populations
Source: PLoS One. 2018 Sep 20;13(9):e0204459. doi: 10.1371/journal.pone.0204459 (PMC6157818; doi:10.1371/journal.pone.0204459)
Supplement: S4 Table — RA: rheumatoid arthritis, OR: odds ratio, CI: confidence interval. Association was tested between the RA patients and the controls by Logistic regression analysis. (PDF) [file pone.0204459.s005.pdf]

S4 Table. Conditional logistic regression analysis of *HLA-DPB1* alleles in the RA patients and controls.

	Uncoditioned				Conditioned on *DRB1* alleles			
Class II allele	OR	95%CI	*P*	*P*c	OR	95%CI	*P*	*P*c
*DPB1*02:01*	1.17	(0.98–1.40)	0.0801	NS	1.39	(1.14–1.70)	0.0014	0.0183
*DPB1*02:02*	0.95	(0.65–1.40)	0.8050	NS	1.03	(0.66–1.60)	0.9125	NS
*DPB1*03:01*	0.92	(0.63–1.35)	0.6860	NS	0.81	(0.53–1.24)	0.3307	NS
*DPB1*04:01*	0.66	(0.46–0.93)	0.0183	0.2378	1.01	(0.60–1.70)	0.9717	NS
*DPB1*04:02*	1.47	(1.12–1.92)	0.0048	0.0629	1.09	(0.78–1.53)	0.6175	NS
*DPB1*05:01*	0.95	(0.81–1.11)	0.4853	NS	0.82	(0.69–0.99)	0.0345	0.4491
*DPB1*06:01*	1.49	(0.44–5.09)	0.5229	NS	1.93	(0.53–7.09)	0.3192	NS
*DPB1*09:01*	0.70	(0.54–0.91)	0.0067	0.0877	0.68	(0.42–1.10)	0.1137	NS
*DPB1*13:01*	0.59	(0.34–1.02)	0.0569	0.7392	0.60	(0.33–1.07)	0.0853	NS
*DPB1*14:01*	1.57	(0.74–3.33)	0.2442	NS	1.15	(0.50–2.62)	0.7485	NS
*DPB1*17:01*	0.58	(0.15–2.24)	0.4260	NS	0.58	(0.07–4.96)	0.6201	NS
*DPB1*19:01*	1.62	(0.36–7.19)	0.5290	NS	0.95	(0.20–4.44)	0.9454	NS
*DPB1*41:01*	1.24	(0.27–5.68)	0.7816	NS	1.07	(0.20–5.63)	0.9407	NS

RA: rheumatoid arthritis, OR: odds ratio, CI: confidence interval. Association was tested between the RA patients and the controls by Logistic regression analysis.
